# Supplementary material for: Solvent-Free Coupling Reaction of Carbon Dioxide and Epoxides Catalyzed by Quaternary Ammonium Functionalized Schiff Base Metal Complexes under Mild Conditions
Source: Materials (Basel). 2023 Feb 16;16(4):1646. doi: 10.3390/ma16041646 (PMC9961927; doi:10.3390/ma16041646)
Supplement: Supplementary file 1 [file materials-16-01646-s001.zip › materials-2133356-supplementary.pdf]

materials

MDPI

Table S1. Provenance and mass fraction purity of the materials.

Materials	Mass fraction	CAS	Provenance
Propylene oxide	0.99	16088-62-3	Shanghai Darui Finechemical Co., Ltd.
1,2-Epoxyethylbenzebe	0.99	96-09-3	Shanghai Darui Finechemical Co., Ltd.
Epichlorihydrin	0.99	106-89-8	Shanghai Darui Finechemical Co., Ltd.
Carbon dioxide	0.9999	124-38-9	Sichuan Tianyi Science & Technology Co., Ltd.
Zinc acetate dihydrate	0.99	5970-45-6	Sinopharm Chemical Reagent Co., Ltd.
Copper(II) acetate monohydrate	0.98	6046-93-1	Sinopharm Chemical Reagent Co., Ltd.
Lead(II) acetate trihydrate	0.995	6080-56-4	Tianjin TEDA letai Chemical Co., Ltd
Cobalt(II) chloride hexahydrate	0.99	7791-13-1	Tianjin Tianda Chemical Co., Ltd.
Nickel(II) chloride hexahydrate	0.98	7791-20-0	Shanghai Shanpu Chemical Co., Ltd.

1. Characterization data for ligands

Table S2. Structure of ligands.

Ligand/ Catalyst	Structure
**NPCl**	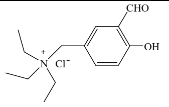
**NPBr**	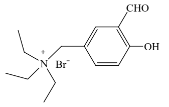
**NPI**	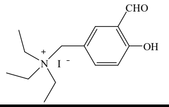

NPCl: 75% yield. 1H NMR (400 MHz, DMSO) δ 11.51 (s,1H),10.32 (s,1H),7.77 (s,1H),7.61 (d,J = 8.4 Hz,1H), 7.25 (d,J = 9.0 Hz,1H),4.46 (s,2H), 3.11 (t,J = 20.2 Hz,6H),1.27 (dd,J = 20.3,13.6 Hz,9H). ^{13}C NMR (101 MHz, DMSO) δ 190.47 , 162.69 , 140.26 , 132.42 , 127.53, 122.33, 118.57, 51.79, 45.17, 7.95. Selected IR peaks (KBr, cm^{-1}): ν2911, 1691, 1601, 1441, 795. Anal. Calcd for $C_{14}H_{22}ClNO_2$: C, 61.87; H, 8.16; N, 5.15%. Found: C, 61.88; H, 8.14; N, 5.12%.

NPBr: 50% yield.^1H NMR (400 MHz, DMSO) δ 11.19 (s, 1H), 10.29 (s, J = 7.3 Hz, 1H), 7.79 (s, J = 16.7, 8.8 Hz, 1H), 7.61 (d, J = 8.6Hz, 1H), 7.11 (d, J = 11.0 Hz, 1H), 4.45 (s, 2H), 3.12 (t, J = 14.1Hz, 6H), 1.28 (dd, 9H).^{13}C NMR (101 MHz, DMSO) δ 190.54 , 163.51, 140.40, 133.44, 123.17, 119.25, 118.53, 52.34, 46.90, 7.97.Selected IR peaks (KBr,cm^{-1}): ν2909,1671,1599,1432,793. Anal. Calcd for $C_{14}H_{22}BrNO_2$: C, 53.17; H, 7.01; N, 4.43%. Found: C, 53.15; H, 7.05, N 4.42%.

NPI: 43% yield. ^1H NMR (400 MHz, DMSO) δ 10.39(s, 1H), 10.11 (s, 1H), 7.77 (s, J = 5.5 Hz, 1H), 7.59 (d, J = 16.7 Hz, 1H), 7.17 (d, 1H), 4.46 (s, J = 8.6 Hz, 2H), 3.09 (t, J = 13.5 Hz, 6H), 1.27 (dd, J = 15.9, Hz, 9H).^{13}C NMR (101 MHz, DMSO) δ 190.52, 162.66, 139.66, 136.19, 132.99, 122.93, 118.05, 51.80, 45.69, 7.75. Selected IR peaks (KBr, cm^{-1}): ν2901, 1662, 1590, 1428, 782. Anal. Calcd for $C_{14}H_{22}INO_2$: C, 46.29; H, 6.10; N, 3.86%. Found: C, 46.28; H, 6.11, N 3.88%.

2. Syntheses of metal complexes Zn-NPClR, Zn-NPXH, M-NPClH

Table S3. Structure of catalysts.

Catalyst	Structure
**Zn-NPClH**	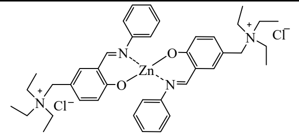
**Zn-NPClCl**	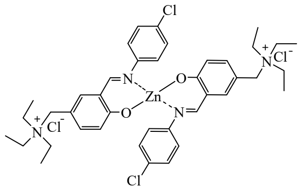
**Zn-NPClNO$_2$**	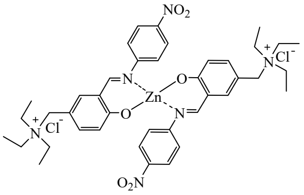
**Zn-NPClCH$_3$**	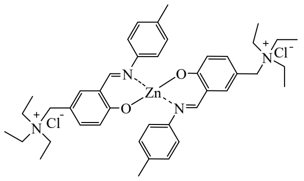
**Zn-NPClC$_4$H$_9$**	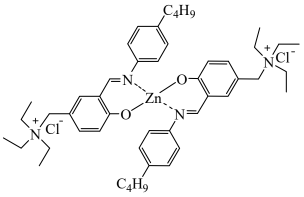
**Zn-NPBrH**	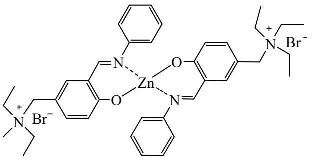
**Zn-NPIH**	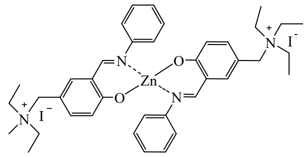
**Co-NPClH**	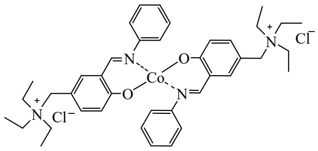
**Pb-NPClH**	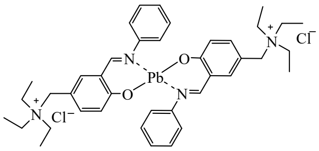
**Ni-NPClH**	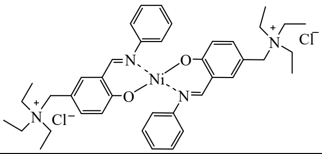

**Cu-NPClH**	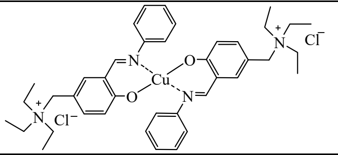

Zn-NPClH: 91% yield. Mp. 204-205 °C;^{1}H NMR (400 MHz, DMSO) δ 8.60 (s,2H),7.64 (d,J = 7.8 Hz,4H),7.48 (dd,J = 15.8,7.9 Hz,6H),7.34 (d,J = 8.1 Hz,4H),6.70 (d,J = 9.2 Hz,2H),4.30 (s,4H),3.18 – 3.09 (m,12H),1.30 (t,J = 6.9 Hz,18H);^{13}C NMR (101 MHz, DMSO) δ 172.04, 168.89, 149.48, 142.43, 138.77, 129.74, 127.27, 123.77, 122.50, 119.32, 112.16, 59.70, 51.71, 49.08, 46.18, 40.57, 40.36, 40.15, 39.94, 39.73, 39.53, 39.32, 10.19, 7.94; Selected IR peaks (KBr,cm^{-1}): ν 3047,1616,1534,1472,1438,691,521;Anal. Calcd for $C_{40}H_{50}Cl_2N_6O_6Zn$: C,63.45; H,6.92; N,7.40%. Found: C,63.36; H,6.96; N,7.48%.

Zn-NPClCl: 78% yield. Mp.78200-201 °C;^{1}H NMR (400 MHz,DMSO) δ 8.59 (s,2H),7.67 (d,J = 8.0 Hz,4H),7.56 – 7.49 (m,6H),7.34 (d,2H),6.69 (d,2H),4.30 (s,4H),3.13 (d,J = 7.0 Hz,12H),1.30 (t,J = 6.7 Hz,18H); ^{13}C NMR (101 MHz,DMSO) δ 172.23,169.18,148.35,142.50,138.93,131.60,129.65,124.28,123.86,119.24,112.26,59.86,51.80,7.95;Selected IR peaks (KBr,cm^{-1}): ν 3069,1617,1582,1532,1468,694,526;Anal.Calcd for $C_{40}H_{50}Cl_4N_4O_2Zn$: C,58.16; H,6.10; N,6.78%. Found: C,58.04; H,6.31; N,6.72%.

Zn-NPClNO$_2$: 90% yield. Mp.208-209 °C;^{1}H NMR (400 MHz,DMSO) δ 8.69 (s,2H),8.36 (d,4H),7.90 – 7.79 (m,4H),7.55 (s,2H),7.39 (d,2H),6.73 (d,2H),4.32 (s,4H),3.14 (d,J = 7.2 Hz,12H),1.30 (t,J = 7.2 Hz,18H);^{13}C NMR (101 MHz,DMSO) δ 171.74,169.62,144.91,141.82,138.56,125.56,125.27,124.31,123.05,122.63,118.09,111.54,110.91, 58.58,50.71,6.87;

Selected IR peaks (KBr,cm^{-1}): ν 3008,1621,1511,1533,1470,694,521;Anal.Calcd for $C_{40}H_{50}Cl_2N_6O_6Zn$: C,56.71; H,5.95; N,9.92%. Found: C,56.58; H,5.98; N,9.81%.

Zn-NPClCH$_3$: 87% yield. Mp.200-201°C;^{1}H NMR (400 MHz,DMSO) δ 8.59 (s,2H),7.67 (d,J = 8.0 Hz,4H),7.56 – 7.49 (m,6H),7.34 (d,2H),6.69 (d,2H),4.30 (s,4H),3.13 (d,J = 7.0 Hz,12H),1.30 (t,J = 6.7 Hz,18H);^{13}C NMR (101 MHz,DMSO) δ 173.09,166.52,145.31,142.55,136.43,126.34,124.25,124.00,122.53,121.89,117.29,110.66,109.32, 57.50,56.56,49.41,6.87;

Selected IR peaks (KBr,cm^{-1}): ν 3069,1617,1582,1532,1468,694,526;Anal.Calcd for $C_{42}H_{56}Cl_2N_4O_2Zn$: C,64.24; H,7.19; N,7.14%. Found: C,64.14; H,7.26; N,7.09%.

Zn-NPClC$_4$H$_9$: 82% yield. Mp.207-208 °C; ^{1}H NMR (400 MHz,DMSO) δ 8.56 (s,2H),7.62 – 7.42 (m,6H),7.39 – 7.14 (m,6H),6.68 (d,2H),4.29 (s,4H),3.12 (d,12H), 2.33 (s,6H),1.30 (t,18H);

^{13}C NMR (101 MHz,DMSO) δ 171.69,169.04,161.64,155.55,142.28,137.07,129.52,122.56,122.28,118.42,116.69,102.68,99.36,55.37,51.70,49.06,32.31,31.16,26.09,20.94,14.23,7.92;Selected IR peaks (KBr,cm^{-1}): ν 3003,1617,1533,1509,1471,658,499; Anal. Calcd for $C_{48}H_{68}Cl_2N_4O_2Zn$: C,66.31; H,7.88; N,6.44%. Found: C,66.26; H,7.96; N,6.29%.

Zn-NPBrH: 89% yield. Mp. 200-203 °C; ^{1}H NMR (400 MHz, DMSO) δ 8.59 (s, 1H), 7.65 (d, J = 7.7 Hz, 2H), 7.52 (s, 1H),7.33(d,3H), 7.08 (d, J = 8.3 Hz, 1H), 6.70 (d, J = 8.8 Hz, 1H), 4.29 (s, 2H), 3.17 – 3.08 (m, 6H), 1.28 (t, J = 7.1 Hz, 9H).^{13}C NMR (101 MHz, DMSO) δ 169.01, 159.90, 148.88, 142.48, 129.90, 127.29, 123.77, 122.61, 121.84, 117.25, 113.32, 113.20, 112.42, 51.79, 46.07, 7.95.Selected IR peaks (KBr,cm-1): 2984,1614,1530,1499,1468,655,496;Anal. Calcd for $C_{48}H_{68}Br_2N_4O_2Zn$: C,56.78; H,6.19; N,6.62%. Found: C,56.70; H,6.23; N,6.55%.

Zn-NPIH: 86% yield. Mp. 198-200 °C; ^{1}H NMR (400 MHz, DMSO) δ 8.64 (d, J = 50.9 Hz, 1H), 7.56 (d, J = 18.0 Hz, 1H), 7.47 (s, 1H), 7.35 (s, 3H), 7.29 – 7.18 (m, 2H), 6.75 (s, 1H), 4.31 (s, 2H), 2.97 (d, J = 131.6 Hz, 6H), 1.38 (t, 9H).13C NMR (101 MHz, DMSO) δ 163.21, 154.45, 142.76, 142.48, 120.89, 119.98, 117.32, 116.81, 115.04, 110.99, 109.32, 109.20, 107.34, 51.81, 46.47, 7.93.

Selected IR peaks (KBr,cm-1): 2978,16014,1523,1496,1459,653,488;Anal.Calcd for C48H68Br2N4O2Zn: C,51.11; H,5.58; N,5.96%. Found: C,51.12; H,5.63; N,5.80%.

Co-NPClH: 88% yield. Mp.233-234 °C; Selected IR peaks (KBr, cm^{-1}): ν 3024,1632,1532,1477,1454,684,505; Anal. Calcd for $C_{40}H_{50}Cl_2N_6O_6Co$: C,57.15; H, 5.99; N, 10.00%. Found: C,57.02; H, 6.10; N, 9.89%.

Pb-NPClH: 43% yield. Mp.210-212 °C; Selected IR peaks (KBr, cm^{-1}): ν 3063,1615,1526,1505,1486,690,510; Anal. Calcd for $C_{40}H_{50}Cl_2N_6O_6Pb$: C, 48.58; H,5.10; N,8.50%. Found: C,48.67; H, 5.21; N, 8.42%.

Ni-NPClH: 36% yield. Mp. 229-231 °C; Selected IR peaks (KBr, cm^{-1}): ν 3006,1615,1506,1485,1455,691,511;Anal.Calcd for $C_{40}H_{50}Cl_2N_6O_6Ni$: C,57.16; H,6.00; N,10.00%. Found: C,57.11; H,6.17; N,9.88%.

Cu-NPClH: 89% yield. Mp.206-208 °C; Selected IR peaks (KBr, cm^{-1}): ν 3053,1616,1532,1507,1466,681,503;Anal.Calcd for $C_{40}H_{50}Cl_2N_6O_6Cu$: C,56.83; H,5.96; N,9.94%. Found: C,56.82; H,5.99; N,9.84%.

3. General procedure for the cycloaddition of CO_2 to epoxides at high pressure

A typical procedure for the coupling reaction of CO_2 and epoxide was as following: A 250 mL stainless steel autoclave with catalyst and stir was dried in vacuum, then it was linked to CO_2 cylinders and purged with CO_2 for three times under atmosphere pressure. When the epoxide was added into the reactor with a hypodermic syringe, the autoclave was sealed and dipped into a pre-heated oil bath at the desired temperature under stirring. Then, carbon dioxide was introduced into the reactor to the set pressure to start the reaction. After the set time, the reaction vessel was cooled quickly with an ice water to release the pressure slowly. The yield and selectivity were obtained by ^{1}H NMR analysis. For all the experiments with different catalysts, no byproduct was detected.

4. General procedure for the cycloaddition of CO_2 to epoxides at atmospheric pressure

A sealed Schlenk flask (75 mL) with catalyst and a magnetic stir linking with a CO_2 balloon was purged with CO_2 for three times. Under the protection of CO_2, epoxide was injected into the flask via syringe, and then the Schlenk flask was dipped into a pre-heated oil bath at a desired temperature and stir started. Then CO_2 was introduced into the system and the reaction time was counted. After the reaction, the yield and selectivity were tested by ^{1}H NMR.

5. Catalyst recycling

For each run, the catalyst was used directly for the next cycle after simple procedure: filtration, washing with acetone and drying in vacuum.

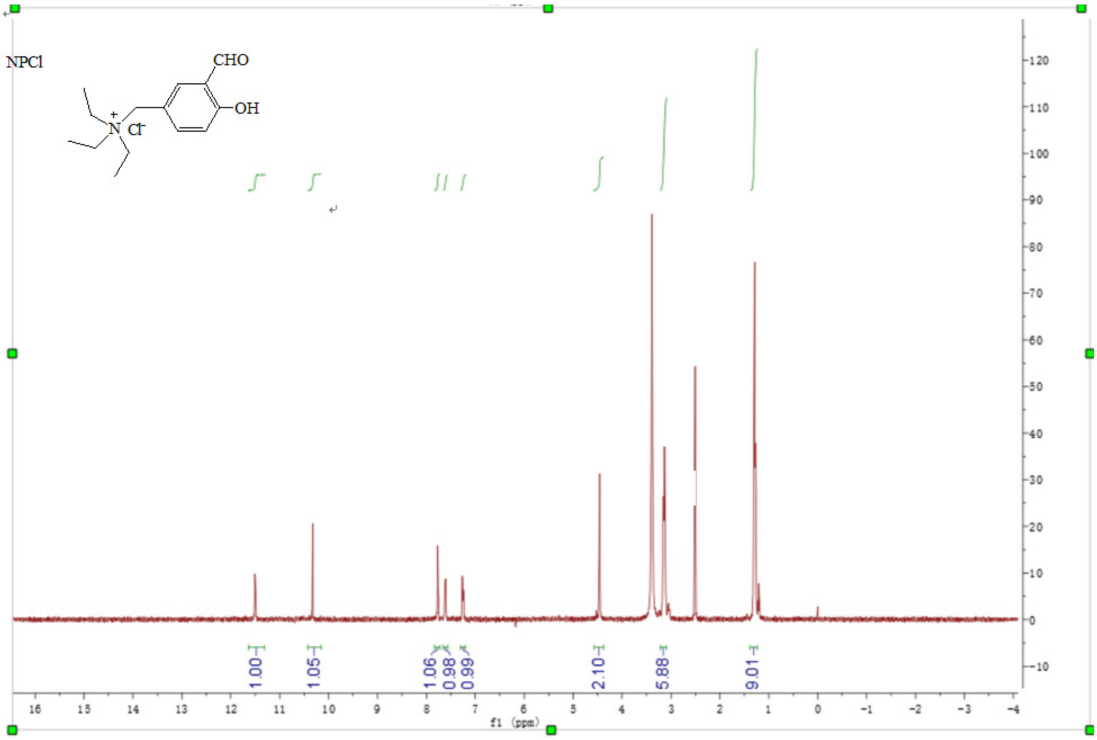

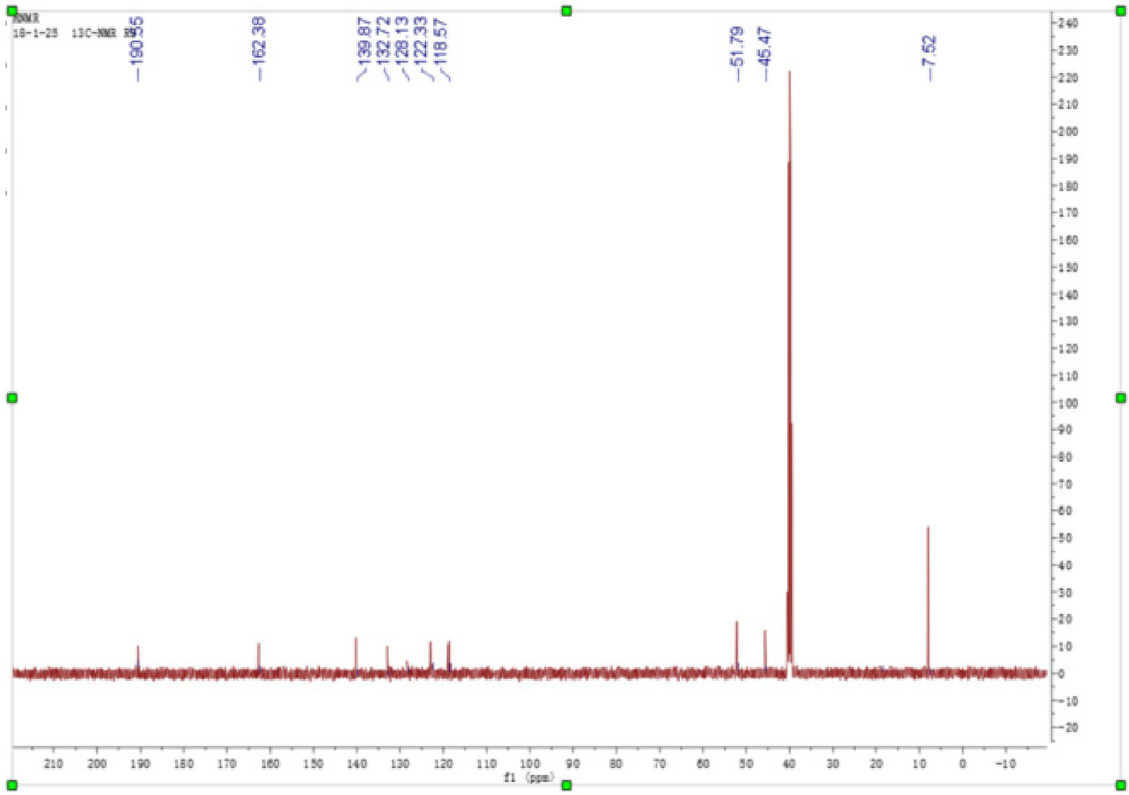

Figure S1. ^{1}H NMR and ^{13}C NMR spectra for **NPCl** in DMSO-d6.

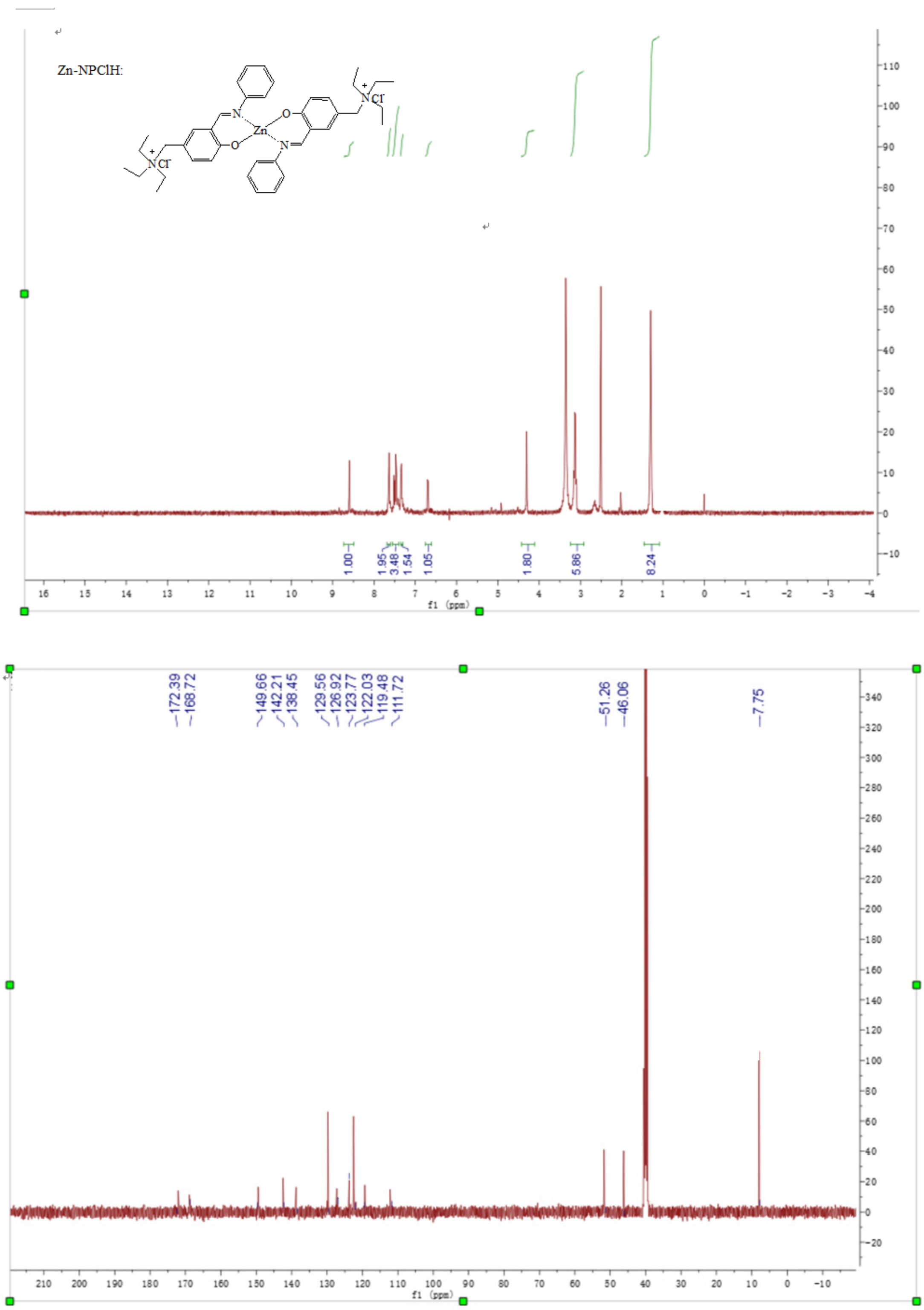

Figure S2. ^{1}H NMR and ^{13}C NMR spectra for **Zn-NPClH** in DMSO-d6.

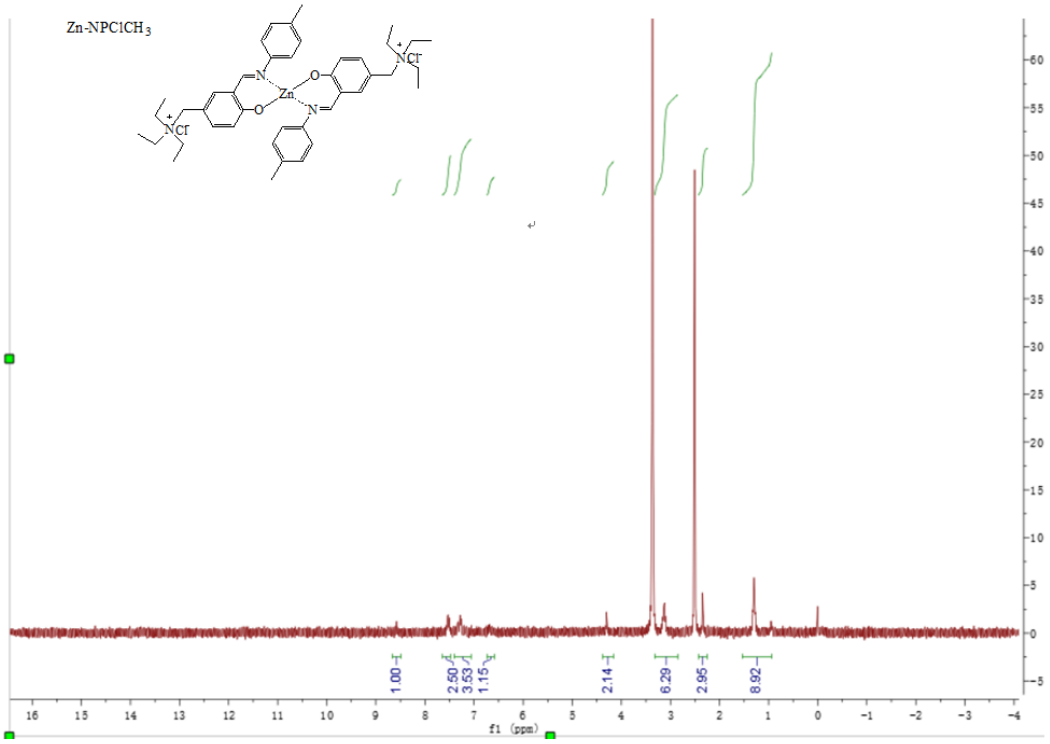

Figure S3. ^{1}H NMR spectrum for **Zn-NPClCH₃** in DMSO-d6.

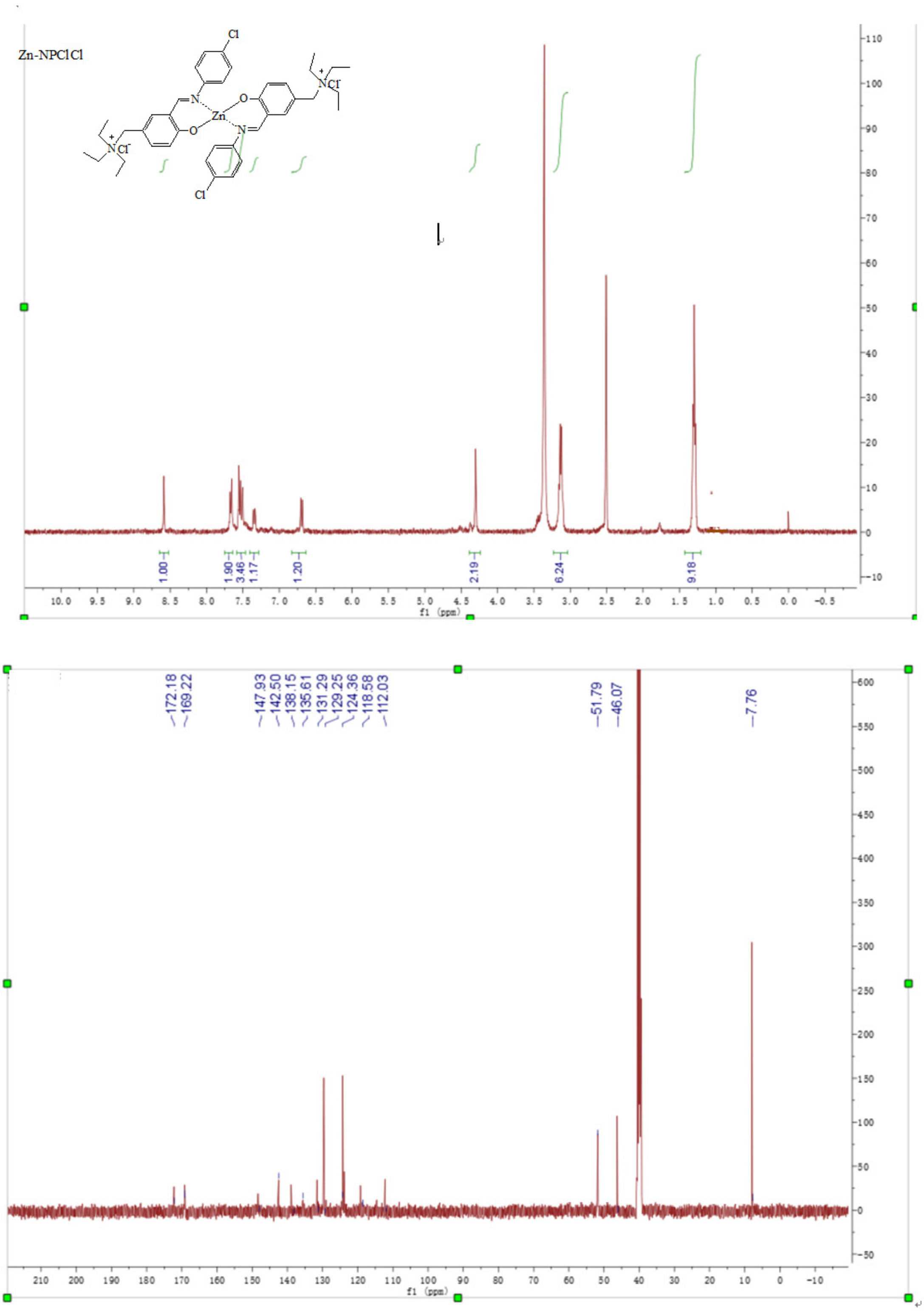

Figure S4. ^{1}H NMR and ^{13}C NMR spectra for **Zn-NPClCl** in DMSO-d6.

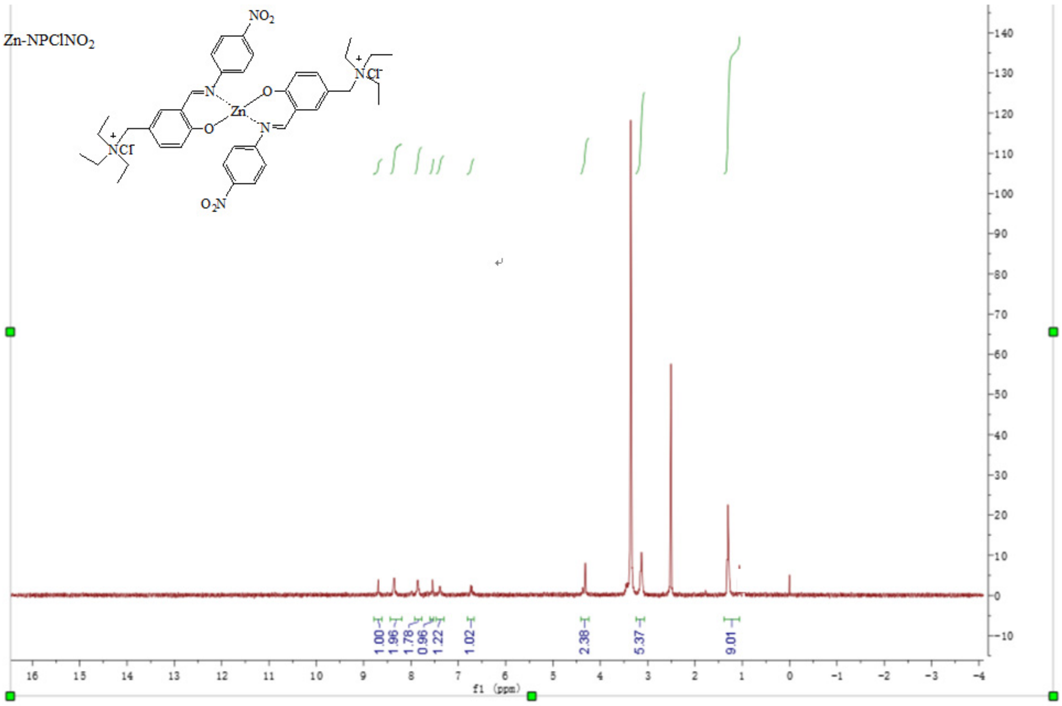

Figure S5. ^{1}H NMR spectrum for **Zn-NPClNO₂** in DMSO-d6.

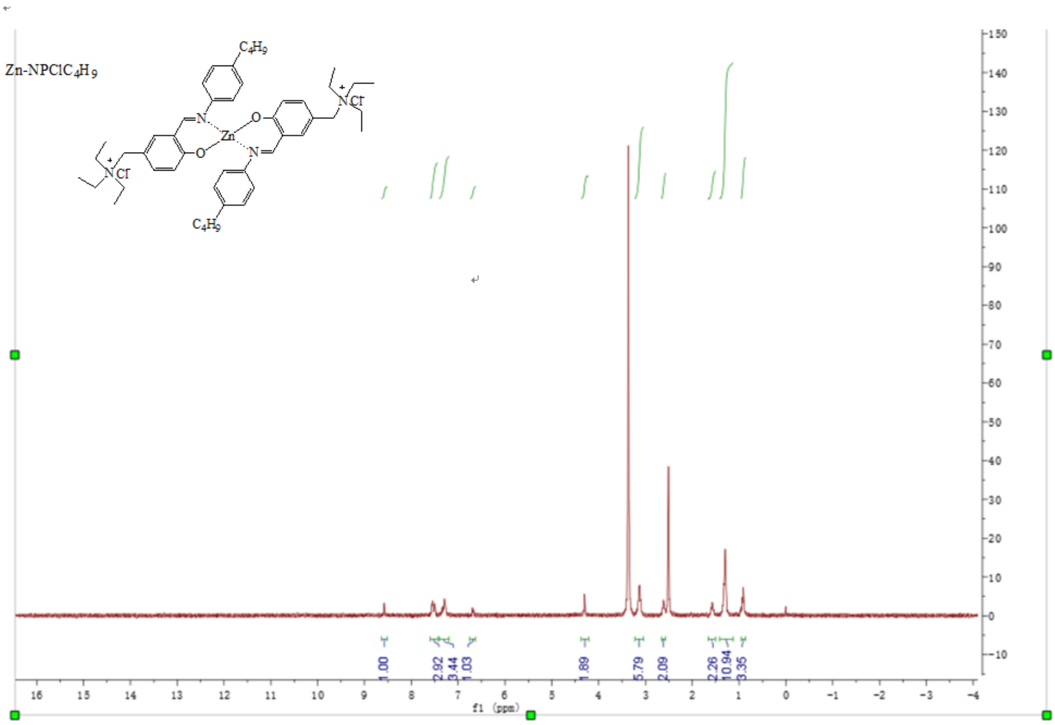

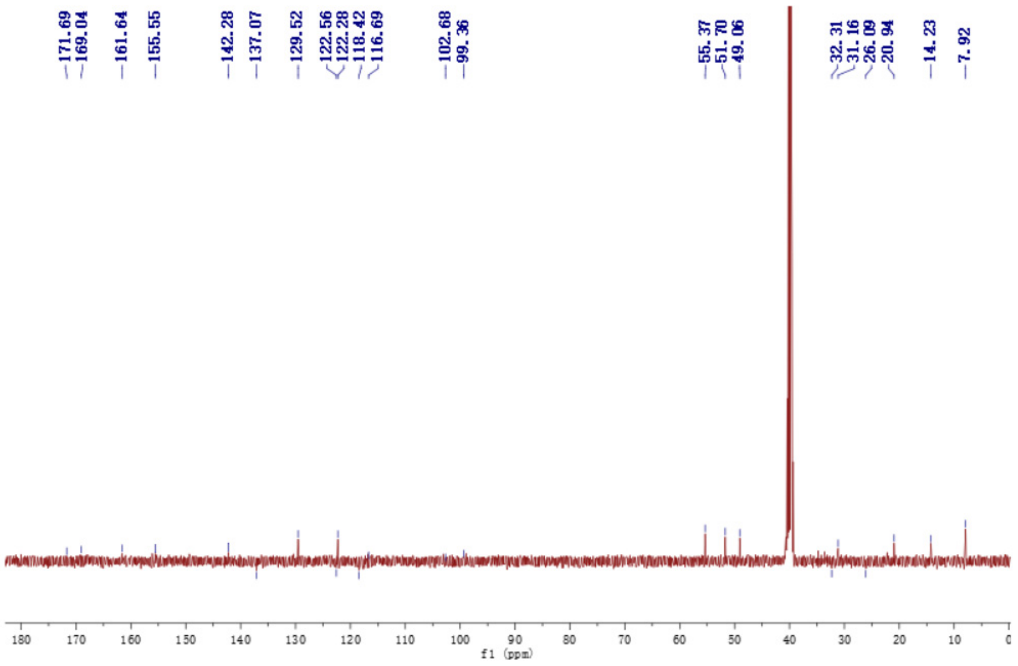

Figure S6. ^{1}H NMR and ^{13}C NMR spectra for **Zn-NPClC4H9** in DMSO-d6.

Table S4. Effects of the reaction time on cycloaddition of CO_2 and glycidyl isopropyl ether at atmospheric pressure by **Zn-NPClH** at 363 K[a].

entry	time (h)	yield (%)[b]	selectivity (%)[b]
1	2	0.03	>99
2	4	0.083	>99
3	6	0.156	>99
4	8	0.264	>99
5	10	0.34	>99

[a] Catalyst: Glycidyl isopropyl ether: 5 mL, **Zn-NPClH**: 1 mol%; CO_2 pressure: 0.1 MPa; temperature: 90 °C.

[b] Yields and selectivities were determined by ^{1}H NMR spectrum of the reaction mixture.

Table S5. Effects of the reaction time on cycloaddition of CO_2 and glycidyl isopropyl ether at atmospheric pressure by **Zn-NPClH** at 373 K[a].

entry	time (h)	yield (%)[b]	selectivity (%)[b]
1	2	0.043	>99
2	4	0.106	>99
3	6	0.239	>99
4	8	0.335	>99
5	10	0.45	>99

[a] Catalyst: Glycidyl isopropyl ether: 5 mL, **Zn-NPClH**: 1 mol%; CO_2 pressure: 0.1 MPa; temperature: 100 °C.

[b] Yields and selectivities were determined by ^{1}H NMR spectrum of the reaction mixture.

Table S6. Effects of the reaction time on cycloaddition of CO_2 and glycidyl isopropyl ether at atmospheric pressure by **Zn-NPClH** at 383 K[a].

entry	time (h)	yield (%)[b]	selectivity (%)[b]
1	2	0.05	>99
2	4	0.165	>99
3	6	0.277	>99
4	8	0.424	>99
5	10	0.53	>99

[a] Catalyst: Glycidyl isopropyl ether: 5 mL, **Zn-NPClH**: 1 mol%; CO_2 pressure: 0.1 MPa; temperature: 110 °C.

[b] Yields and selectivities were determined by ^{1}H NMR spectrum of the reaction mixture.

Table S7. Effects of the reaction time on cycloaddition of CO_2 and glycidyl isopropyl ether at atmospheric pressure by **Zn-NPClH** at 393 K[a].

entry	time (h)	yield (%)[b]	selectivity (%)[b]
1	2	0.056	>99
2	4	0.212	>99
3	6	0.372	>99
4	8	0.507	>99
5	10	0.597	>99

[a] Catalyst: Glycidyl isopropyl ether: 5 mL, **Zn-NPClH**: 1 mol%; CO_2 pressure: 0.1 MPa; temperature: 120 °C.

[b] Yields and selectivities were determined by ^{1}H NMR spectrum of the reaction mixture.

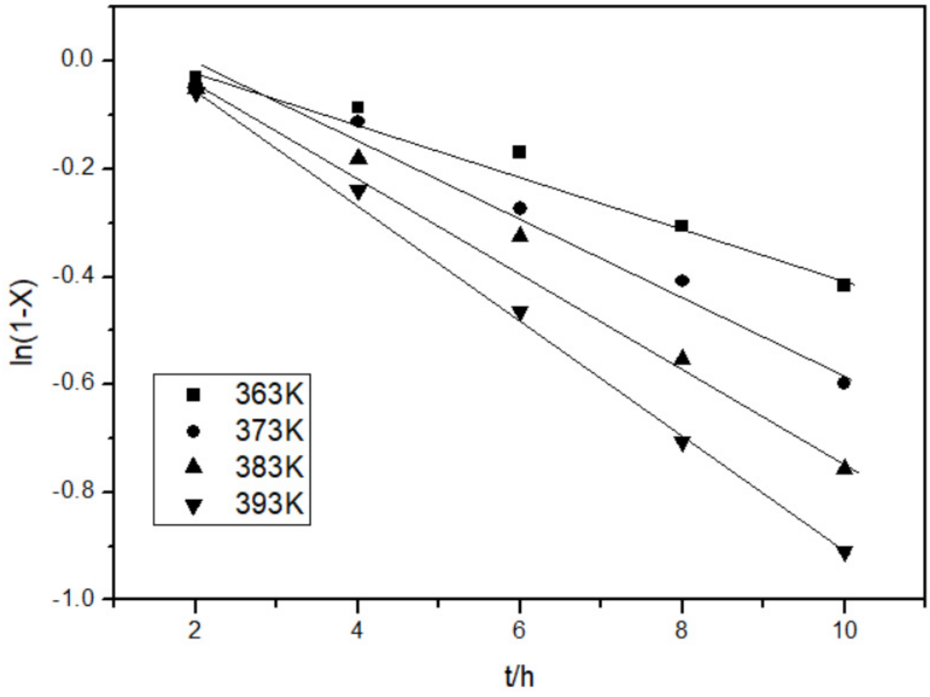

Figure S7. Logarithmic plots of $(1 - x)$ versus time by **Zn-NPClH** at 363–393 K.

Table S8. Effects of the reaction time on cycloaddition of CO_2 and glycidyl isopropyl ether at atmospheric pressure by **Co-NPClH** at 363 K[a].

entry	time (h)	yield (%)[b]	selectivity (%)[b]
1	2	0.063	>99
2	4	0.185	>99
3	6	0.326	>99
4	8	0.417	>99
5	10	0.489	>99

[a] Catalyst: Glycidyl isopropyl ether: 5 mL, **Co-NPClH**: 1 mol%; CO_2 pressure: 0.1 MPa; temperature: 90 °C.

[b] Yields and selectivities were determined by ^{1}H NMR spectrum of the reaction mixture.

Table S9. Effects of the reaction time on cycloaddition of CO_2 and glycidyl isopropyl ether at atmospheric pressure by **Co-NPClH** at 373 K[a.]

entry	time (h)	yield (%)[b]	selectivity (%)[b]
1	2	0.082	>99
2	4	0.233	>99
3	6	0.405	>99
4	8	0.523	>99
5	10	0.583	>99

[a] Catalyst: Glycidyl isopropyl ether: 5 mL, **Co-NPClH**: 1 mol%; CO_2 pressure: 0.1 MPa; temperature: 100 °C.

[b] Yields and selectivities were determined by ^{1}H NMR spectrum of the reaction mixture.

Table S10. Effects of the reaction time on cycloaddition of CO_2 and glycidyl isopropyl ether at atmospheric pressure by **Co-NPClH** at 383 K[a.]

entry	time (h)	yield (%)[b]	selectivity (%)[b]
1	2	0.104	>99
2	4	0.295	>99
3	6	0.487	>99
4	8	0.591	>99
5	10	0.68	>99

[a] Catalyst: Glycidyl isopropyl ether: 5 mL, **Co-NPClH**: 1 mol%; CO_2 pressure: 0.1 MPa; temperature: 110 °C.

[b] Yields and selectivities were determined by ^{1}H NMR spectrum of the reaction mixture.

Table S11. Effects of the reaction time on cycloaddition of CO_2 and glycidyl isopropyl ether at atmospheric pressure by **Co-NPClH** at 393 K[a.]

entry	time (h)	yield (%)[b]	selectivity (%)[b]
1	2	0.125	>99
2	4	0.33	>99
3	6	0.51	>99
4	8	0.64	>99
5	10	0.73	>99

[a] Catalyst: Glycidyl isopropyl ether: 5 mL, **Co-NPClH**: 1 mol%; CO_2 pressure: 0.1 MPa; temperature: 120 °C.

[b] Yields and selectivities were determined by ^{1}H NMR spectrum of the reaction mixture.

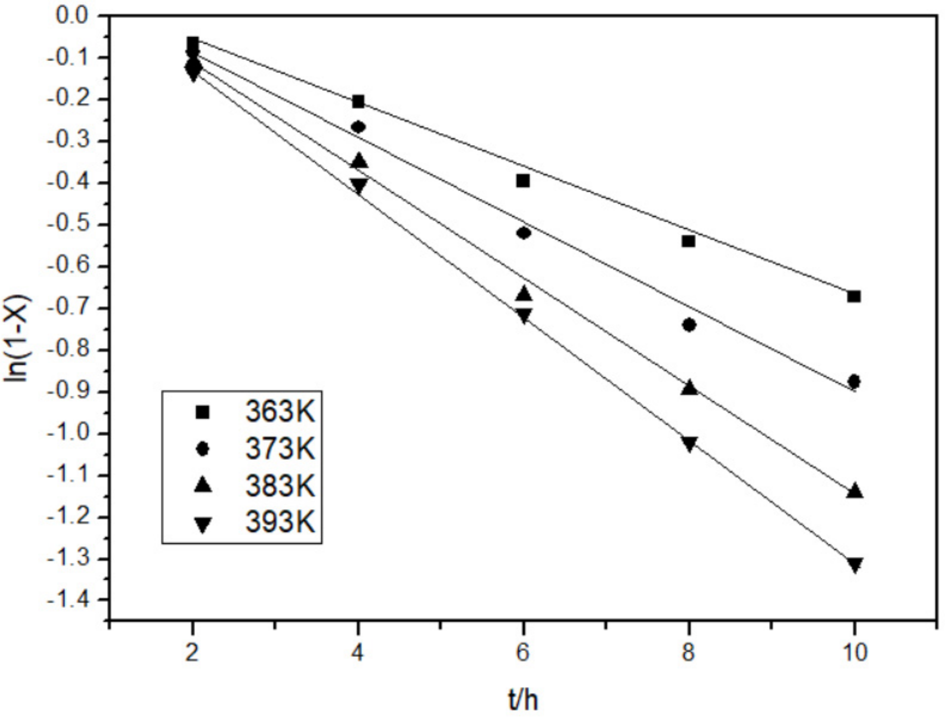

Figure S8. Logarithmic plots of $(1 - x)$ versus time by **Co-NPClH** at 363–393 K.

Table S12. Effects of the reaction time on cycloaddition of CO_2 and glycidyl isopropyl ether at atmospheric pressure by **Ni-NPClH** at 363 K[a.]

entry.	time (h)	yield (%)[b]	selectivity (%)[b]
1	2	0.02	>99
2	4	0.03	>99
3	6	0.06	>99
4	8	0.08	>99
5	10	0.12	>99

[a] Catalyst: Glycidyl isopropyl ether: 5 mL, **Ni-NPClH**: 1 mol%; CO_2 pressure: 0.1 MPa; temperature: 90 °C.

[b] Yields and selectivities were determined by ^{1}H NMR spectrum of the reaction mixture.

Table S13. Effects of the reaction time on cycloaddition of CO_2 and glycidyl isopropyl ether at atmospheric pressure by **Ni-NPClH** at 373 K[a].

entry	time (h)	yield (%)[b]	selectivity (%)[b]
1	2	0.01	>99
2	4	0.05	>99
3	6	0.08	>99
4	8	0.14	>99
5	10	0.18	>99

[a] Catalyst: Glycidyl isopropyl ether: 5 mL, **Ni-NPClH**: 1mol%; CO_2 pressure: 0.1 MPa; temperature: 100 °C.

[b] Yields and selectivities were determined by ^{1}H NMR spectrum of the reaction mixture.

Table S14. Effects of the reaction time on cycloaddition of CO_2 and glycidyl isopropyl ether at atmospheric pressure by **Ni-NPClH** at 383 K[a].

entry	time (h)	yield (%)[b]	selectivity (%)[b]
1	2	0.01	>99
2	4	0.06	>99
3	6	0.1	>99
4	8	0.15	>99
5	10	0.21	>99

[a] Catalyst: Glycidyl isopropyl ether: 5 mL, **Ni-NPClH**: 1 mol%; CO_2 pressure: 0.1 MPa; temperature: 110 °C.

[b] Yields and selectivities were determined by ^{1}H NMR spectrum of the reaction mixture.

Table S15. Effects of the reaction time on cycloaddition of CO_2 and glycidyl isopropyl ether at atmospheric pressure by **Ni-NPClH** at 393 K[a.]

entry	time (h)	yield (%)[b]	selectivity (%)[b]
1	2	0.03	>99
2	4	0.06	>99
3	6	0.11	>99
4	8	0.17	>99
5	10	0.26	>99

[a] Catalyst: Glycidyl isopropyl ether: 5 mL, **Ni-NPClH**: 1 mol%; CO_2 pressure: 0.1 MPa; temperature: 120 °C.

[b] Yields and selectivities were determined by ^{1}H NMR spectrum of the reaction mixture.

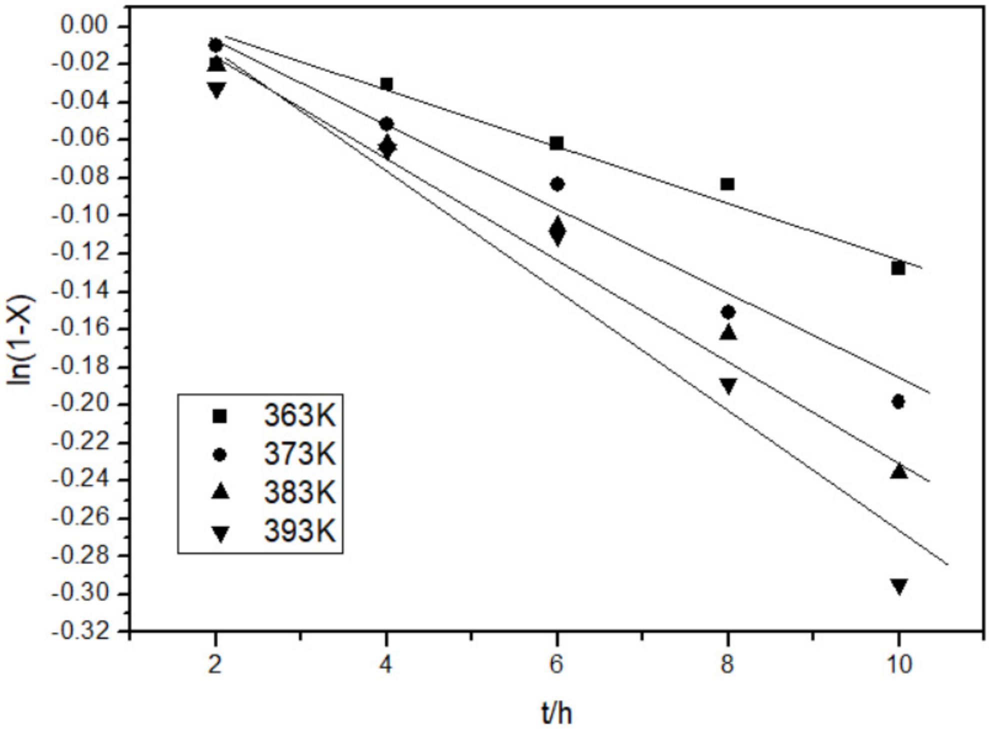

Figure S9. Logarithmic plots of (1 – x) versus time by **Ni-NPClH** at 363–393 K.
